# Detection of reactive oxygen species in isolated, perfused lungs by electron spin resonance spectroscopy

**DOI:** 10.1186/1465-9921-6-86

**Published:** 2005-07-31

**Authors:** Norbert Weissmann, Nermin Kuzkaya, Beate Fuchs, Vedat Tiyerili, Rolf U Schäfer, Hartwig Schütte, Hossein A Ghofrani, Ralph T Schermuly, Christian Schudt, Akylbek Sydykov, Bakytbek Egemnazarow, Werner Seeger, Friedrich Grimminger

**Affiliations:** 1 Justus-Liebig University, Department of Internal Medicine II, Klinikstrasse 36, 35392 Giessen, Germany; 2 Charite, Department of Internal Medicine, Humboldt-University, 13353 Berlin, Germany; 3 ALTANA Pharma, 78467 Konstanz, Germany

## Abstract

**Background:**

The sources and measurement of reactive oxygen species (ROS) in intact organs are largely unresolved. This may be related to methodological problems associated with the techniques currently employed for ROS detection. Electron spin resonance (ESR) with spin trapping is a specific method for ROS detection, and may address some these technical problems.

**Methods:**

We have established a protocol for the measurement of intravascular ROS release from isolated buffer-perfused and ventilated rabbit and mouse lungs, combining lung perfusion with the spin probe l-hydroxy-3-carboxy-2,2,5,5-tetramethylpyrrolidine (CPH) and ESR spectroscopy. We then employed this technique to characterize hypoxia-dependent ROS release, with specific attention paid to NADPH oxidase-dependent superoxide formation as a possible vasoconstrictor pathway.

**Results:**

While perfusing lungs with CPH over a range of inspired oxygen concentrations (1–21 %), the rate of CP^• ^formation exhibited an oxygen-dependence, with a minimum at 2.5 % O_2_. Addition of superoxide dismutase (SOD) to the buffer fluid illustrated that a minor proportion of this intravascular ROS leak was attributable to superoxide. Stimulation of the lungs by injection of phorbol-12-myristate-13-acetate (PMA) into the pulmonary artery caused a rapid increase in CP^• ^formation, concomitant with pulmonary vasoconstriction. Both the PMA-induced CPH oxidation and the vasoconstrictor response were largely suppressed by SOD. When the PMA challenge was performed at different oxygen concentrations, maximum superoxide liberation and pulmonary vasoconstriction occurred at 5 % O_2_. Using a NADPH oxidase inhibitor and NADPH-oxidase deficient mice, we illustrated that the PMA-induced superoxide release was attributable to the stimulation of NADPH oxidases.

**Conclusion:**

The perfusion of isolated lungs with CPH is suitable for detection of intravascular ROS release by ESR spectroscopy. We employed this technique to demonstrate that 1) PMA-induced vasoconstriction is caused "directly" by superoxide generated from NADPH oxidases and 2) this pathway is pronounced in hypoxia. NADPH oxidases thus may contribute to the hypoxia-dependent regulation of pulmonary vascular tone.

## Background

Reactive oxygen species (ROS) play an important role in biological systems. While it is largely accepted that ROS-mediated oxidative damage occurs under pathophysiological conditions, recent evidence also favors a role for ROS as signaling molecules in physiological processes [1-6]. In the respiratory system, ROS are involved in pathological states such as hyperoxia-induced lung injury, sepsis and ischemia-reperfusion, but may also play a role in the development of hypoxia-and non-hypoxia-induced pulmonary hypertension [7-9]. In the context of regulatory processes of the lung, a growing body of evidence is emerging indicating that ROS may contribute to signaling events such as underlying hypoxic pulmonary vasoconstriction (HPV), an essential mechanism matching lung perfusion to ventilation in order to optimize pulmonary gas exchange [10,11]. The question of whether ROS generation is decreased or paradoxically increased during alveolar hypoxia, and the sources from which ROS may be derived under these conditions, are controversial [8,12]. In fact, substantial evidence exists suggesting both decreases as well as increases, in lung ROS generation during alveolar hypoxia [13-15]. Both mitochondria and NADPH oxidases have been proposed as source(s) of ROS generation underlying HPV [12,13,15-21].

One possible reason for the discrepancies among the different concepts of hypoxia-dependent ROS generation in the lung may be the inadequacies of the methods applied. For example, most of the evidence provided for increased ROS generation on the cellular level is based on measurements with fluorochromes, such as dihydrorhodamin 123 or 2',7'-dichlorofluorescin diacetate, which have been shown to be ROS generators themselves under various conditions [22,23]. In isolated perfused lungs, luminol and lucigenin, cytochrome c reduction, as well as spin trapping with 5,5-dimethyl-l-pyrrorine-*N*-oxide or sodium 3,5-dibromo-4-nitrosobenzenesulfonate [14,24] have been used for detection of ROS. These methods are, however, also prone to pitfalls due to autoxidation of the substrates, artificial ROS generation by redox cycling [22,24,25] or the fact that the product interferes with ascorbate in the tissue [26]. The ROS measurements have also been performed in rabbit lungs with the spin probe sodium 3,5-dibromo-4-nitrosobenzenesulfonatesodium [27]. However, in this investigation, the spin probe was added to the effluent of the lung, which did not allow detection of ROS release directly at its source in the lung vasculature [27].

To overcome several of these problems and to investigate in particular the role of hypoxia in NADPH oxidase-derived superoxide release and its role in the regulation of pulmonary vascular tone, we established a method combining ESR spectroscopy with the spin trapping technique for measurement of superoxide release from isolated perfused and ventilated rabbit and mouse lungs. Superoxide was detected by the cyclic hydroxylamine l-hydroxy-3-carboxy-2,2,5,5-tetramethylpyrrolidine (CPH) which was recently introduced for quantitative ROS measurements in biological systems [28]. Employing this agent, ROS formation can be quantified by detection of the corresponding nitroxide radical 3-carboxy-proxyl (CP^•^) by ESR spectroscopy [29,30]. After having proven the feasibility of CPH for ROS detection in the isolated perfused and ventilated rabbit lungs, with particular attention being paid to the autoxidation of CPH, we investigated the oxygen-dependence of intravascular ROS release in the intact lungs. Moreover, we quantified CPH oxidation over a range of inspired oxygen concentrations, while stimulating NADPH oxidases with phorbol-12-myristate-13-acetate (PMA). These experiments were performed in the absence and presence of superoxide dismutase (SOD) to estimate the proportion of superoxide-induced CPH oxidation. We thus demonstrated the feasibility of the ESR technique, and obtained interesting new insights into the oxygen-dependence of baseline versus PMA/NADPH oxidase-dependent superoxide generation in the intact lung vasculature.

## Methods

### Chemicals and reagents

l-hydroxy-3-carboxy-2,2,5,5-tetramethylpyrrolidine (CPH) was purchased from L-Optik (Berlin, Germany). Krebs-Henseleit buffer contained 125.0 mM NaCl, 4.3 mM KC1, 1.1 mM KH_2_PO_4_, 2.4 mM CaCl_2_, 1.3 mM MgCl_2 _and 275 mg glucose per 100 ml. FeCl_2_, deferoxamine (DFO), diethyldithiocarbamate (DETC), phorbol-12-myristate-13-acetate (PMA), and Cu/Zn-superoxide dismutase (SOD) were obtained from Sigma (Deisenhofen, Germany). Apocynin was from Merck Biosciences (Schwalbach, Germany).

### In-vitro CPH experiments

For studying CPH characteristics and the effect of metal chelating agents on background ESR signals, experiments were performed in glass tubes containing 1 mM CPH solution prepared in Krebs-Henseleit buffer at room temperature. The iron chelator DFO was added in the concentrations 20 μM or 2 mM to the solution and measurements were run every 30 min for a total period of 5 h.

### Isolated rabbit lung experiments

The technique of isolated lung perfusion and ventilation has been described previously in detail [31,32]. Briefly, pathogen-free New Zealand white rabbits of either sex (body weight 2.5–3.2 kg) were deeply anesthetized with ketamine (30–50 mg/kg body weight) and xylazine (6–10 mg/kg body weight), and anticoagulated with heparin (1000 U/kg body weight). The lungs were excised while perfused with Krebs-Henseleit buffer through cannulas in the pulmonary artery and the left atrium. After the lungs were rinsed with at least 1 1 of buffer fluid for removal of blood, the perfusion circuit was closed for recirculation (total system volume 150 ml) and left venous pressure set at 2 mmHg to secure West zone III conditions for perfusion. In parallel with the onset of artificial perfusion, room air ventilation was changed to a mixture of 5.3 % CO_2_, 21.0 % O_2 _and the balance N_2 _(tidal volume, 30 ml; frequency, 30 strokes/min) with the use of positive end-expiratory pressure of 1 cm H_2_O. The pH was adjusted to 7.35 – 7.40 by addition of NaHCO_3_. The isolated lungs were placed in a temperature-equilibrated housing chamber and the whole system was heated to 38.5°C.

Krebs-Henseleit buffer was incubated with 5 μM diethyldithiocarbamate overnight to allow sedimentation. Briefly before the experiments, 20 μM DFO and NaHCOs were added to the supernatant.

Lungs included in the study were those that i) had a homogeneous white appearance with no signs of hemostasis, edema or atelectasis, ii) revealed constant mean pulmonary artery and peak ventilation pressure in the normal range, and iii) were isogravimetric during the initial perfusion period of 55 min.

For normoxic and hypoxic ventilation maneuvers, a gas-mixing chamber (KM 60-3/6 MESO, Witt, Witten, Germany) was employed for step changes in the ventilator O_2 _content. 5.3 % CO_2 _were used throughout and the percentage of N_2 _was balanced accordingly.

After the initial steady state period with normoxic ventilation (21 % O_2_), CPH (1 mM) was added to the buffer fluid. Five minutes later, one of three different protocols was initiated:

1) A 2.5 or 3.0 h period of normoxic ventilation with 21 % O_2_, in the case of a 3 h ventilation period followed by a bolus application of PMA into the pulmonary artery, resulting in a concentration of 1 μM in the recirculating buffer fluid. Control experiments received a bolus of saline instead of PMA,

2) Consecutive 30 min periods of ventilation with different O_2 _concentrations (21, 16, 10, 5, 2.5 or 1 % O_2 _in a randomized fashion) for a total period of 3 h, or

3) One 30 min-period of ventilation at either 21, 16, 10, 5, 2.5 or 1 % O_2_, followed by a bolus application of PMA into the pulmonary artery, resulting in a concentration of 1 μM in the recirculating buffer fluid. Control experiments received a bolus of NaCl instead of PMA.

In a portion of the experiments a fiber oxygenator (Hilite 1000, Stolberg, Germany) was used instead of the lung for oxygenation of the buffer fluid.

### Isolated mouse lung experiments

Mouse lung experiments were performed in a protocol analogous to the isolated rabbit lung experiments but in an in-chest preparation as previously described [33]. Lungs were perfused with 0.5 mM CPH at a flow rate of 2.0 ml/min for 120 min at normoxic ventilation (21 %O_2_), followed by a bolus application of PMA into the buffer fluid, resulting in a concentration of 10 μM. For these investigations either C57/BL6 mice (= wildtype control) or mice lacking the NADPH oxidase subunit gp91phox (= p91phox^-/-^). Mice were obtained from The Jackson Laboratory (Bar Harbor, Maine, USA).

### ESR measurements

Oxidation of the spin probe CPH by superoxide forms the nitroxide CP radical. The triple-line spectrum of CP radical was detected by ESR spectroscopy, using a MS 100 spectrometer (Magnettech, Berlin, Germany). The ESR measurements were performed in field scan with the following settings: microwave frequency 9.78 GHz, modulation frequency 100 kHz, modulation amplitude 2 G, microwave power 18 mW. All samples, both from the in vitro experiments or from the venous outflow of the isolated lung, were made in 50 μl glass capillary tubes and measured immediately at room temperature. The ESR amplitude is in proportion to the amount of CP^•^, reflecting the interaction of ROS with CPH [29,34,35]. Thus, the quantity of trapped ROS was directly calculated from the ESR spectrum of the probe, while the contribution of superoxide radical to the formation of CP was determined in parallel experiments performed in the presence of SOD in the buffer fluid (150 U/ml). The first sample was taken 5 min after CPH addition to the buffer fluid (time set at zero), followed by further sampling every 5 or 30 minutes, as appropriate. The values after PMA addition were assessed every minute in the respective experiments. In the isolated mouse lung samples were taken every 2 min. For quantification, the second-field component of the ESR spectrum was used. To standardize values, the amplitude of this component was divided through the receiver gain.

### Statistical analysis

Data are given as mean ± standard error (SEM). For comparison of two groups, a two-tailed t-test was employed. For multiple comparisons, analysis of variance was used, followed by the Student-Newman-Keuls post hoc test when differences were indicated. Statistical significance was assumed when p < 0.05.

## Results

**Figure 1:**
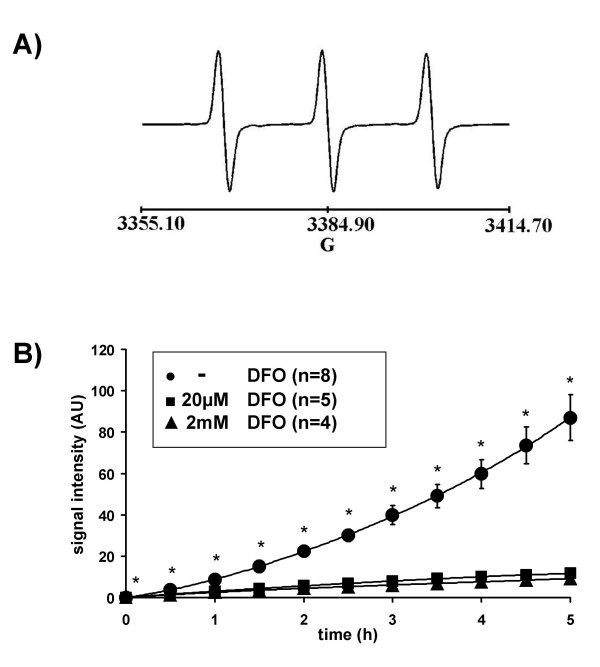
**The effect of the iron chelating agent deferoxamine (DFO) on CP^• ^nitroxide signal intensity in vitro. **(A) Typical ESR spectrum of CP^• ^nitroxide resulting from the reaction of the hydroxylamine spin probe CPH with ROS. The height of the first field component of the triple-line spectrum was used for quantification of signal intensity. (B) In-vitro incubation of CPH (1 mM) in Krebs-Henseleit buffer. Signal intensity is given in arbitrary units (AU). Data are shown for CPH oxidation in the absence (-DFO) or in the presence of either 20 μM or 2 mM deferoxamine (DFO). Asterisks indicate significant differences when compared to the-DFO group.

**Figure 2:**
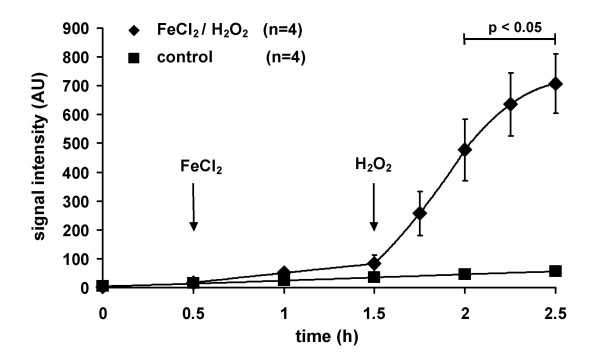
**The ESR signal intensity in isolated perfused and ventilatedrabbit lungs during baseline conditions and in the presence of FeCl_2_/H_2_O_2_. **Lungs were either perfused with Krebs-Henseleit buffer containing 1 mM CPH only (control) or in the presence of FeCl_2 _(1 μM, added at 0.5 h, FeCl_2_/H_2_O_2_). After 1.5 h, H_2_O_2 _was added to the buffer fluid by continuous infusion (8 μmol/min) in the FeCl_2_-perfused lungs. Bar indicates significant differences between the FeCl_2_/H_2_O_2 _group and control.

When CPH (1 mM) was dissolved in Krebs-Henseleit buffer ESR spectroscopy resulted in a triple band spectrum (Fig. 1A). The ESR signal increased to 87.06 ± 11.17 arbitrary units, (AU, n = 8) within 5 h when incubated in-vitro at room temperature (Fig. 1B). The presence of the iron chelating agent DFO at a concentration of 20 μM or 2 mM, from the beginning of the experiment, reduced the ESR amplitude ~8-fold to 11.66 ± 0.44 (n = 5) or 9.28 ± 0.57 (n = 4) AU within 5 h, with no major difference being observed between the high and the low concentration of DFO (Fig. 1B). Pre-incubation of the buffer with the copper chelator DETC (5 μM) further reduced the increase in signal (data not shown). In line with these in vitro data, ESR signal intensity increased when isolated rabbit lungs were perfused under normoxic ventilation with CPH (1 mM) for 2.5 h (Fig. 2). Perfusion with FeCl_2 _(1 μM) resulted in a markedly higher ESR signal intensity (Fig. 2). Addition of H_2_O_2 _to the buffer fluid (8 μmol/min, started after 1.5 h), in the presence of FeCl_2_, induced a strong increase in ESR signal intensity, reaching a value of 707.14 ± 103.15 AU (n = 4) within 1 h of lung perfusion (Fig. 2).

**Figure 3:**
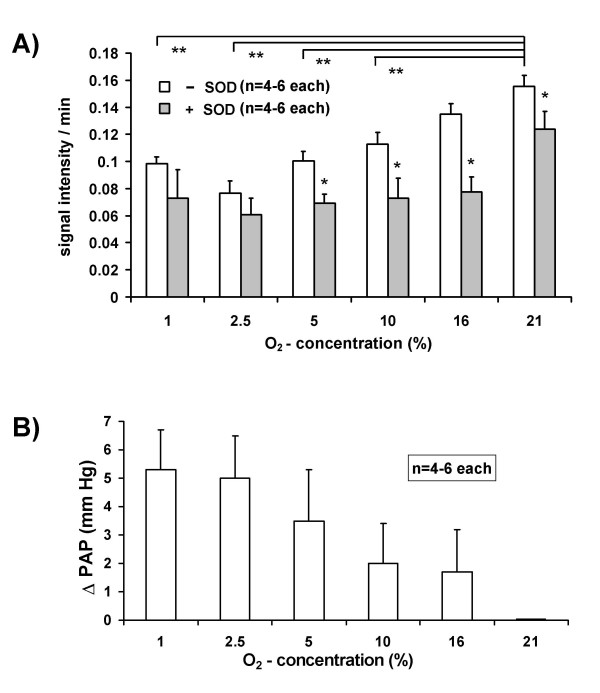
**Hypoxia-dependent superoxide release and vasoconstrictor responses in isolated perfused and ventilated rabbit lungs. **During a total 3 h period of perfusion, the ventilator gas supply was switched to different oxygen concentrations every 30 min, using 1 %, 2.5 %, 5 %, 10 %, 16 % or 21 % O_2 _in a randomized mode. (A) Superoxide release. The rate of increase in ESR signal intensity turned out to be linear during the last 20 min of each ventilation period. Therefore, changes in the ESR signal intensity/min during the last 20 min of each ventilation period are given. In the +SOD group, 150 mU/ml superoxide dismutase (SOD) was present throughout the experiments. * significant differences between the +SOD and the -SOD group for the respective oxygen concentration. ** significant differences as compared to 21 % O_2_. (B) Vasoconstrictor response. The maximum increases in PAP (ΔPAP) are given for the different oxygen concentrations and are referenced to baseline (=normoxic) PAP values, which were 8.8 ± 0.6 mmHg

Performing a sequential mode of 30 min periods of ventilation maneuvers with different inspiratory oxygen concentrations (1 % – 21 % O_2_) revealed oxygen-dependent changes in ESR signal intensity. The ESR signals demonstrated a linear increase during the last 20 min of each maneuver with samples taken every 5 min. This time-dependent increase in the ESR signal intensity was highest when ventilating the lungs with 21 % O_2 _and lowest when ventilating the lungs with 2.5 % O_2 _(Fig. 3A). There was a tendency towards increased values found when the lungs were ventilated with 1 % O_2_, compared with 2.5 % O_2_. Parallel experiments in the presence of SOD (150 U/ml), revealed that a portion of the signal was derived from superoxide. Figure 3B depicts the effect of the ventilation maneuvers with the different oxygen concentrations on pulmonary artery pressure (PAP). Hypoxic ventilation induced an increase in PAP, which was highest at 1 % and lowest at 16 % O_2_.

**Figure 4:**
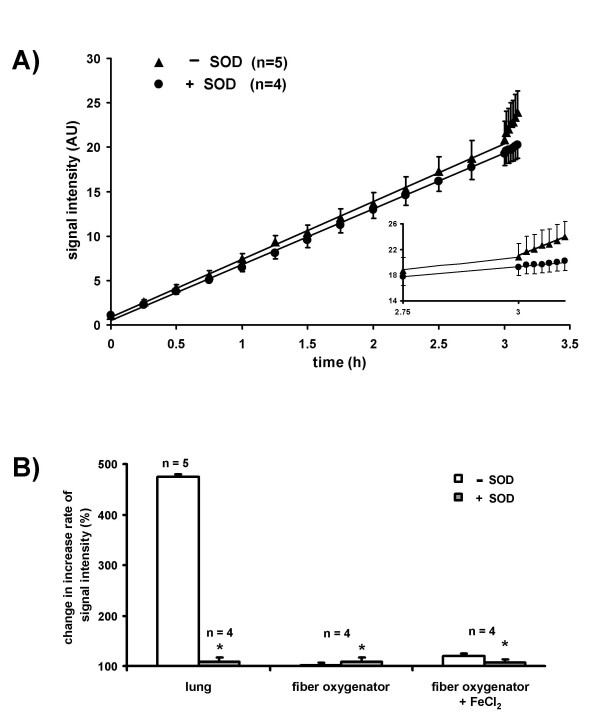
**Superoxide release from isolated perfused rabbit lungs afteraddition of phorbol-12-myristate-13-acetate (PMA) during normoxic ventilation. **(A) ESR signal intensity during normoxic ventilation of rabbit lungs in the presence (+SOD) and the absence of SOD (-SOD). After 3 h, PMA was injected into the pulmonary artery, resulting in a concentration of 1 μM in the recirculating buffer fluid. The increase in ESR signal intensity was linear before and after addition of PMA. The insert shows the PMA effect with higher time resolution. (B) Changes in the increase rate of the ESR signal intensity (%) by comparison of values prior to and after addition of PMA to isolated rabbit lungs. In the +SOD group, SOD was present throughout the experiments. In two separate sets of experiments, a fiber oxygenator was used instead of the lung for oxygenation of the recirculating buffer fluid ("fiber oxygenator"). The fibre oxygenator experiments were performed either in the absence ("fiber oxygenator") or in the presence of 1 μM FeCl_2 _("fiber oxygenator + FeCl_2_") in the buffer fluid. * significant difference between the +SOD and the -SOD group

In Fig. 4A, the time-dependent increase of CP^• ^formation is illustrated for long-term normoxic ventilation (3 h, 21% O_2_) of isolated lungs. After 3 h, a bolus of PMA, resulting in a concentration of 1 μM in the recirculating buffer fluid, was injected into the pulmonary artery. This application induced a rapid increase in the ESR signal intensity, which was almost entirely blocked by the addition of SOD. As the time-dependent increase in ESR signal intensity was linear before and after addition of PMA, increase rates, pre-and post-intervention, may be directly compared (Fig. 4B). While almost fully suppressed by SOD, a five-fold increase in CP^• ^formation per unit time occurred in response to the PMA stimulation. For controls, we performed corresponding experiments, in which the lung was replaced by a fiber oxygenator, in order to exclude a direct effect of PMA on CPH oxidation. No response to PMA was observed in the absence of the lung vasculature either in the presence or in the absence of 1 μM FeCl_2 _(Fig. 4B).

**Figure 5:**
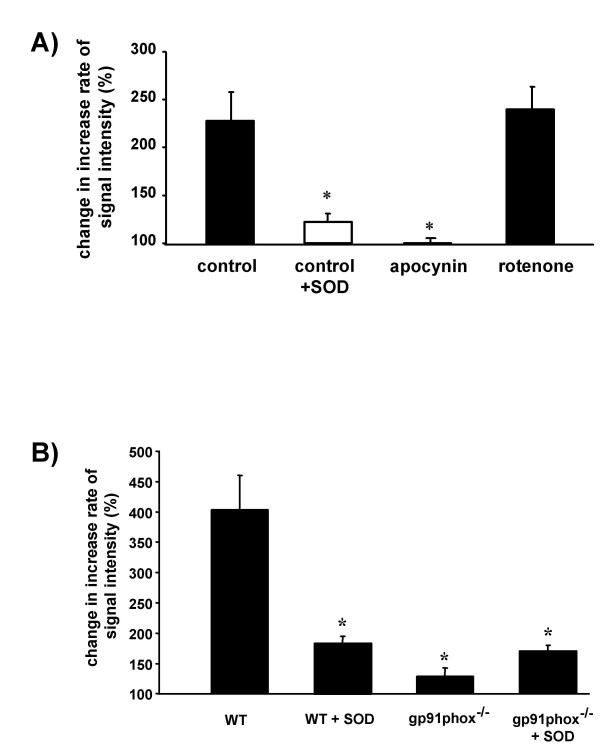
**Effects of the NADPH oxidase and mitochondrial inhibitors, as well as of phagocytic NADPH oxidase gene deletion on the ESR signal intensity in isolated perfused lungs after addition of phorbol-12-myristate-13-acetate (PMA) during normoxic ventilation. **(A) Effect of the NADPH oxidase inhibitor apocynin (500 μM) and the inhibitor of mitochondrial complex I, rotenone (350 nM) on the increase rate in ESR signal intensity after addition of PMA. Each inhibitor was added to the buffer fluid 30 min before addition of PMA. In the +SOD group, SOD was present throughout the experiments. * significant difference as compared to control. (B) Comparison of the increase rate in ESR signal intensity after addition of PMA in wildtype (WT) and gp91phox-deficient (gp91phox^-/-^) mice. Lungs were perfused for 120 min prior to PMA addition (10 μM) either in the presence or absence of 150 U/ml SOD. * significant difference as compared to WT. Data are given as changes in the increase rate of the ESR signal intensity after PMA addition, as compared to the values before PMA addition (set as 100%). Data are from n = 4–5 experiments for each group.

The PMA-induced increase in ESR signal intensity in the isolated lung experiments was inhibited by the NADPH oxidase inhibitor apocynin but not by rotenone, an inhibitor of mitochondrial complex I (Fig. 5A). Moreover, in mice lacking the NADPH oxidase subunit gp91phox, the PMA-induced increase in the ESR signal intensity was prevented, when compared to wildtype mice (Fig. 5B). Corresponding to the SOD-inhibitable increase in ESR signal intensity in the perfused rabbit lungs, PMA application induced a vasoconstrictor response, which was also largely attenuated in the presence of SOD as well as in the presence of apocynin (table 1).

**Table 1:** Baseline pulmonary artery pressure (PAP) and phorbol-12-myristate-13-acetate (PMA)-induced changes in PAP after 3 hours of normoxic ventilation. Pulmonary artery pressure values (PAP) are given for time points directly before and 6 min after addition of PMA to the buffer fluid. In addition, the increase in PAP per minute (ΔPAP/min) after PMA addition is indicated. Data are shown for experiments in the absence (-SOD) and the presence of 150 U/ml superoxide dismutase (+SOD). The PMA was added after 3 h of normoxic ventilation. Values of the +SOD and -SOD group correspond to experiments in Fig. 4. In the apocynin group this agent was present in the perfusate at a concentration of 500 μM. In experiments without addition of PMA, no significant change in PAP was observed within 6 min after PMA addition. Asterisk indicates significant difference of the -SOD as compared to the +SOD or apocynin group.

	**directly before PMA [mmHg]**	**6 min after PMA**	**ΔPAP/min after PMA**	**n**
-SOD	10.4 ± 0.4	15.2 ± 1.1	0.66 ± 0.18	5
+SOD	8.9 ± 0.7	9.9 ± 0.7	0.15 ± 0.02	4 *
apocynin	8.2 ± 0.4	8.3 ± 1.0	0.03 ± 0.01	4 *

**Figure 6:**
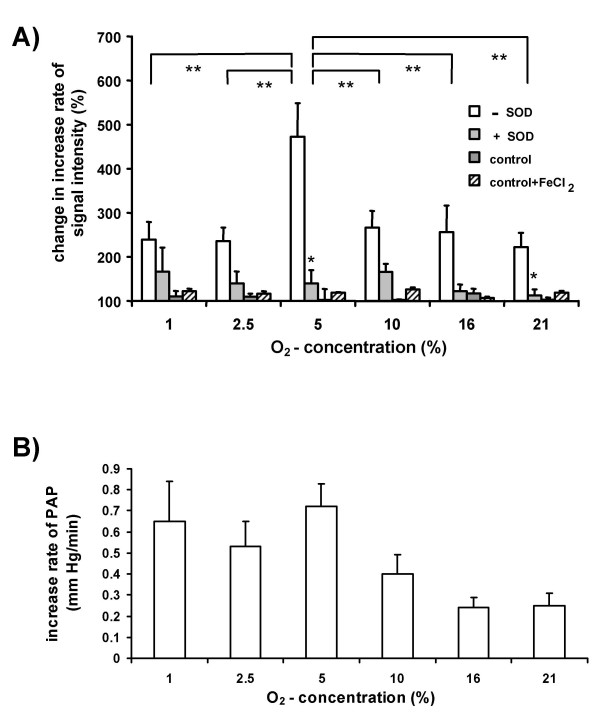
**Oxygen-dependency of PMA-induced changes in ESR signal intensity, lung superoxide release, and pulmonary artery pressure. **Lungs were ventilated for 30 minutes with either 1 %, 2.5 %, 5 %, 10 %, 16 % or 21 % O_2_, followed by injection of PMA into the pulmonary artery, resulting in a concentration of 1 μM in the recirculating buffer fluid. (A) Changes in the increase rate of the ESR signal intensity after PMA addition, as compared to the values before PMA addition (set as 100 %) are given. Experiments were performed in the presence (+SOD) or the absence (-SOD) of SOD. In the control group, lungs were replaced by a fiber oxygenator for equilibration of the buffer fluid with the different oxygen concentrations. The fibre oxygenator experiments were performed either in the absence or in the presence of 1 μM FeCl_2 _in the buffer fluid. * significant differences between +SOD and -SOD groups. ** significant differences between different oxygen concentrations. (B) Increase in pulmonary artery pressure per minute (ΔPAP/min) within 7 min after PMA addition to the buffer fluid. In experiments without addition of PMA no significant change in PAP was observed. Data are from n = 4–9 experiments for each group.

To investigate the effect of inspired oxygen concentration on the PMA-induced increment in CP^• ^formation, we performed short-term ventilation periods of 30 min at varying O_2 _concentrations, followed by bolus application of PMA (1 μM) into the pulmonary artery. The increases in the ESR signal intensity/min post-PMA was compared to that pre-PMA application (Fig. 6A). The highest increase in ESR signal intensity/time was observed when the lungs were ventilated with 5 % O_2_, within a total range of 1 – 21 % O_2_. Parallel experiments in the presence of SOD, revealed that the major portion of the increase in the ESR signal was due to superoxide release. Again, replacement of the lungs by a fiber oxygenator, to equilibrate the buffer fluid with the different oxygen concentrations did not change the increase rate in ESR signal intensity after PMA application, either in the absence or in the presence of 1 μM FeCl_2 _(Fig. 6A). PMA application induced an increase in PAP (Fig. 6B). This increase was highest when lungs were ventilated with 5 % O_2_.

## Discussion

### Methodological aspects

Because ROS formation has been implicated in a variety of lung diseases, the detection of ROS release from the pulmonary circulation is thought to be important [7-9]. However, current methods for the detection of lung vascular ROS formation lack specificity due to autoxidation of the substrates employed, the lack reliability due to artificial ROS generation by redox cycling [22,24,25], and a lack sensitivity of the techniques employed. The ESR technology may overcome several of these shortfalls. In a previous study, Katz and colleagues used spin trapping and ESR technology for ROS detection in isolated perfused rabbit lungs. However, in that investigation, the spin probe was added to the lung effluent, and was therefore unable to detect ROS release at its source within the vasculature, which would be desirable due to the short half-life of ROS [27]. We therefore favored the strategy to add the spin probe directly to the buffer fluid, and employed the cyclic hydroxylamine spin probe CPH, as this is an effective ROS scavenger allowing quantitative measurement of ROS formation [29,30]. Even under conditions of hypoxia-induced vasoconstriction the spin probe is expected to reach all parts of the lungs as these were perfused at constant flow under zone III conditions [31]. The reaction rate constant of CPH with ROS is 3.2 × 10^3 ^M^-1 ^s^-1^, which is approximately 10-fold higher than the reaction rate constant of nitrone spin traps such as DMPO or DEPMPO [30,36]. The incorporation of CPH into the cell membrane is low, permitting analysis of extracellular ROS release, while the high stability of the formed nitroxides allowed quantitative measurement of the reaction product in the lung effluent [30,35].

CPH was, however, reported to be autoxidized through traces of transition metals, such as iron and copper, which can be eliminated by chelators [37]. Therefore, we first characterized CPH properties incubated in vitro in Krebs-Henseleit-buffer, which is routinely used for perfusion of the isolated lungs. Measurement of aliquots from this solution resulted in the typical triple-band spectrum [29,30]. The prominent reduction in the increased ESR signal intensity observed within a five-hour incubation period, in the presence of the iron chelator DFO, confirmed autoxidation of CPH catalyzed by traces of transition metals in the buffer fluid. This interpretation is also supported by the observation that the increase in signal intensity during perfusion of CPH through the lung is further enhanced by the addition of FeCl_2 _to the buffer fluid. No major difference was found between low and high DFO concentrations, and preincubation of the buffer with the copper chelator DETC further reduced CPH autoxidation. We therefore conducted all subsequent experiments in the presence of 20 μM DFO after preincubation of the buffer with DETC (5 μM). The DETC inhibits Cu, Zn -SOD. However, inhibition was only observed when much higher concentrations (>1 mM) were applied compared with the concentration employed in our study [38]. These findings are supported by other investigations from our laboratory, demonstrating that HPV in isolated rabbit lungs is not altered even at DECT concentrations 20 times higher than those employed in the present study [21].

When the DFO-and DETC-treated buffer was employed for perfusion of isolated and ventilated rabbit lungs, we first demonstrated that CPH is suitable for detection of ROS by the admixture of H_2_O_2 _to the buffer fluid, which resulted in a prominent increase in ESR signal intensity. This increase is most likely related to the generation of hydroxyl radicals (OH^•^) via Fenton reaction [39-41]. Although these data demonstrate the suitability of the technique, it became apparent that continuous oxidation of CPH circulating through the lungs occurs, which may depend on ongoing ROS release, or may reflect autoxidation independent of lung ROS liberation.

### Oxygen-dependency of superoxide release

To investigate the dependency of the ESR signal increase on the inspired oxygen concentration, successive 30 min ventilation periods with different O_2 _concentrations of the inspired gas were performed. We found a decreased rate of ESR signal enhancement at lower oxygen concentrations, with a minimum at 2.5 % O_2_, whereas 1 % O_2 _caused again some increase. Addition of SOD to the buffer fluid reduced the rate of ESR signal increase at all oxygen concentrations, proving that at least a portion of the ESR signal detected is caused by intravascular superoxide release from the lungs. Several enzyme systems may be responsible for oxygen-dependent release of superoxide into the lung vasculature, including the NADPH-oxidases, xanthine-oxidase, nitric oxide synthases and arachidonic acid oxygenase pathways, as well as the mitochondrial respiratory chain [9,13,14,20]. Moreover, non-superoxide-derived ROS may add to the baseline increase in CPH oxidation, not suppressed by SOD [35]. As extracellular SOD is not thought to enter the intracellular milieu [42,43], intracellular trapping of superoxide by CPH may further add to this finding. However, as only low penetration of cell membranes was described for CPH this is less likely [34,44,45].

Ventilation of the lungs with oxygen concentrations lower than 21 % resulted in increased pulmonary artery pressure, with the changes in proportion to the relative O_2 _decrease being highest between 10.0 and 2.5 %, as previously reported for isolated rabbit lungs [31]. This hypoxia-induced vasoconstriction is known as a key feature of the lung, matching perfusion to ventilation in order to optimize pulmonary gas exchange by excluding poorly or non-ventilated areas from blood flow. Among other concepts, lung ROS generation has been suggested to be involved in this mechanism [15,46]. However, there is ongoing discussion about whether an increase or a decrease in ROS occurs during hypoxia, and from what source hypoxia-induced ROS may be derived [12,14,17,18,20,21]. As possible sources for both a decrease and an increase, mitochondria or NADPH-oxidases have been suggested [8,12]. With regard to these aspects it has not yet been determined whether hypoxia can indeed cause a paradoxical increase in NADPH oxidase-dependent superoxide release in the pulmonary circulation. The fact that extracellular SOD inhibited the ESR signal, but not the PAP increase under hypoxic conditions [8,32] can be explained by intracellular superoxide production, e.g. in the vascular smooth muscle cells, underlying the mechanism of hypoxic vasoconstriction, but not being a major contributor to the intravascular superoxide leak. Thus, the current focus on the intravascular compartment for ROS trapping may not be a suitable approach for analyzing ROS formation that underlies hypoxic pulmonary vasoconstriction. It may, however, be appropriate to measure ROS formation under conditions primarily affecting the endothelial cells, such as endothelial injury and ischemia-reperfusion conditions. Therefore, we targeted key enzyme systems that may be involved in endothelial ROS generation.

### NADPH oxidase-dependent superoxide release and pulmonary vasoconstriction

To provoke a more pronounced ROS formation in the vascular compartment and to investigate the oxygen-dependence of NADPH oxidase-derived superoxide release, we employed intravascular administration of PMA, which was previously shown to stimulate NADPH oxidases via activation of protein kinase C [27]. Indeed, PMA induced a prominent increase in the rate of CPH oxidation, mostly attributable to intravascular superoxide release, as evident from a total block of this increase in the presence of SOD. Replacement of the lung by a fiber oxygenator to mimic oxygenation of the buffer fluid, as would occur in the lung, assured that no lung-independent oxidation of CPH was provoked by PMA, neither in the absence nor in the presence of FeCl_2_. Thus an overlapping effect of metal ions primarily being responsible for the oxygen-dependent effects seen in the presence of the lung as e.g. results from a Fenton reaction, can be excluded. The PMA-induced increase in the ESR signal was illustrated in our study to be attributable to the suggested pathway of NADPH oxidase stimulation, because it was prevented a) by the NADPH oxidase inhibitor apocynin as well as b) in mice lacking the NADPH oxidase subunit gp91phox (Nox-2). In contrast, rotenone, a mitochondrial complex I inhibitor, did not affect the PMA induced ROS release. This indicated that mitochondria-derived superoxide does not play a role in the oxygen-dependent ROS release induced by PMA. This finding is of particular interest, given the recent reports of mitochondria as possible sources of superoxide release [17]. Moreover, PMA caused an immediate pulmonary artery pressor response, which was also largely blocked by SOD, suggesting a direct vasoconstrictor effect of superoxide generated by PMA addition. This suggestion is in line with the inhibition of the vasoconstrictor response by the NADPH oxidase inhibitor apocynin. The fact that PMA stimulation of the lung induces a vasoconstrictor response via superoxide challenges previous studies suggesting that the PMA-induced vasoconstrictor response involves a Ca^2+ ^sensitization by inhibition of myosin light chain phosphatase (for review see [47]). The superoxide-induced vasoconstriction in this pathway may involve intracellular calcium mobilization by enhancing cyclic ADP-ribose production [48], activation of RhoA/Rho kinase [49], or inactivation of NO [50] by superoxide. To investigate the oxygen-dependence of the PMA-induced superoxide release, we then stimulated the lungs with PMA in the presence of different oxygen concentrations. Most interestingly, we detected peak PMA-evoked lung superoxide release when lungs were ventilated with 5 % O_2_. This peak in superoxide release correlated with the maximum PMA-evoked vasoconstrictor effect. The NADPH oxidases of endothelial cells, which have been shown to contain all NADPH oxidase subunits needed for superoxide generation as well as leukocytes are resident in the intravascular compartment, and are suggested as a possible source of the PMA-induced superoxide release. The ESR technology was not suitable for detecting significant hypoxia-dependent changes in superoxide release in unstimulated isolated rabbit lungs. However, since i) hypoxia caused an increased release of NADPH-dependent superoxide release when lungs were challenged with PMA and ii) that superoxide caused a vasoconstriction; it is tempting to speculate that such mechanisms may contribute to the regulation of HPV. Data from our laboratory have repetitively suggested that an NADPH oxidase-dependent increase in lung ROS release contributes to the initiation of HPV [8,21]. Thus, it is interesting that many studies investigating HPV in isolated lungs the pulmonary circulation was primed with angiotensin II to yield a sufficient hypoxic vasoconstrictor response [51-53]. Angiotensin II has also been shown to activate NADPH oxidases and thus a possible interference of an angiotensin II-induced superoxide release in HPV has to be taken into account. The increase in NADPH oxidase-dependent superoxide release may not only play a role under physiological conditions, but may also contribute to pathophysiological pathways in conditions of ischemia-reperfusion, implicating not only a role of superoxide during reperfusion phase, but also in the ischemic phase.

Three distinct effects have to be taken into consideration regarding the mechanism of PMA-induced increase in intravascular superoxide release. First, a direct effect of hypoxia on the activity of NADPH oxidases. Although such an effect is not known for the NADPH oxidases from phagocytic cells, this may not be excluded for vascular NADPH oxidases that are composed of specific isoforms [54]. Thus, regulation of electron flux through the NADPH oxidase rather than oxygen itself (even at low concentrations) may be the rate-limiting step in superoxide formation. This suggestion is supported by the notion that NADPH oxidase-dependent superoxide release is stimulated by anoxia in cultured aortic endothelial cells [55]. Second, the increased superoxide release may be related to alterations in the activation mechanism upstream of NADPH oxidase (e. g. protein kinase c) or may be related to alterations in protein kinase c-independent mechanisms of NADPH oxidase assembly [55]. Third, alterations to the intravascular or cellular oxygen scavenging capacity may also contribute to an increased superoxide release in hypoxia.

## Conclusion

In summary, we have established a new method to detect intravascular ROS release in intact lungs, combining ESR technology with lung perfusion in the presence of the spin probe CPH. This technique may be useful in elucidating the role of ROS in different physiological and pathophysiological conditions where ROS are suggested to be involved and to correlate ROS release to vasoreactivity. Focusing on hypoxia-dependent ROS release, we demonstrated that i) that PMA-induced vasoconstriction is caused by superoxide generated from NADPH oxidases (rather than by H_2_O_2 _or by a protein kinase c-dependent increase in Ca^2+ ^sensitivity) and ii) that this pathway is pronounced in hypoxia. The NADPH oxidases thus may contribute the hypoxia-dependent regulation of pulmonary vascular tone.

## Abbreviations

AU: arbitrary units

CP^•^: 3-carboxy-proxyl radical

ΔPAP: change in pulmonary artery pressure

DETC: diethyldithiocarbamate

DFO: deferoxamine

ESR: electron spin resonance

HPV: hypoxic pulmonary vasoconstriction

CPH: l-hydroxy-3-carboxy-2,2,5,5-tetramethylpyrrolidine

PAP: pulmonary artery pressure

ROS: reactive oxygen species

PMA: phorbol-12-myristate-13-acetate

SOD: Cu/Zn-superoxide dismutase

## Authors' contributions

Conception and design: N. Weissmann, N. Kuzkaya, H. Schütte, H. A. Ghofrani, R. T. Schermuly, W. Seeger, F. Grimminger.

Analysis and interpretation of the data: N. Weissmann, N. Kuzkaya, R. U. Schäfer, H. Schütte, H. A. Ghofrani, R. T. Schermuly, W. Seeger, F. Grimminger, B. Fuchs, V. Tiyerili, C. Schudt, A. Sydykov, B. Egemnazarow.

Drafting of the article: N. Weissmann, N. Kuzkaya, W. Seeger, F. Grimminger. Critical revision of the article for important intellectual content: N. Weissmann, N. Kuzkaya, H. Schütte, H. A. Ghofrani, R. T. Schermuly, W. Seeger, F. Grimminger, B. Fuchs, V. Tiyerili, A. Sydykov, B. Egemnazarow, C. Schudt, R. U. Schäfer.

Final approval of the article: N. Weissmann, N. Kuzkaya, H. Schütte, H. A. Ghofrani, R. T. Schermuly, W. Seeger, F. Grimminger, B. Fuchs, V. Tiyerili, A. Sydyjov, B. Egemnazarow.

Provision of study materials: N. Weissmann, N. Kuzkaya, H. Schütte, H. A. Ghofrani, R. T. Schermuly, C. Schudt, R. U. Schäfer, W. Seeger, F. Grimminger,.

Statistical expertise: B. Fuchs, V. Tiyerili, A. Sydykov, B. Egemnazarow, N. Weissmann, N. Kuzkaya, R. U. Schäfer.

Performance of experiments: B. Fuchs, V. Tiyerili, A. Sydykov, B. Egemnazarow, N. Weissmann, N. Kuzkaya, R. U. Schäfer.

Obtaining of funding: N. Weissmann, N. Kuzkaya, H. Schütte, W. Seeger, F. Grimminger. Administrative, technical, or logical support: N. Weissmann, N. Kuzkaya, H. Schütte, C. Schudt, W. Seeger, F. Grimminger.

Collection and assembly of data: N. Weissmann, N. Kuzkaya, H. Schütte, H. A. Ghofrani, R.T. Schermuly, W. Seeger, F. Grimminger, B. Fuchs, V. Tiyerili, A. Sydykov, B. Egemnazarow, R. U. Schäfer.

N. Weissmann and N. Kuzkaya contributed equally to this work

Portions of the doctoral thesis of Vedat Tiyerili and Rolf Ulrich Schäfer are incorporated into this report.

## References

[B1] Cai H, Harrison DG (2000). Endothelial dysfunction in cardiovascular diseases: the role of oxidant stress. Circ Res.

[B2] Fukai T, Siegfried MR, Ushio-Fukai M, Cheng Y, Kojda G, Harrison DG (2000). Regulation of the vascular extracellular superoxide dismutase by nitric oxide and exercise training. J Clin Invest.

[B3] Griendling KK, Harrison DG (1999). Dual role of reactive oxygen species in vascular growth. Circ Res.

[B4] Harrison DG (1997). Cellular and molecular mechanisms of endothelial cell dysfunction. J Clin Invest.

[B5] Munzel T, Harrison DG (1997). Evidence for a role of oxygen-derived free radicals and protein kinase C in nitrate tolerance. J Mol Med.

[B6] Sauer H, Wartenberg M, Hescheler J (2001). Reactive oxygen species as intracellular messengers during cell growth and differentiation. Cell Physiol Biochem.

[B7] Midorikawa J, Maehara K, Yaoita H, Watanabe T, Ohtani H, Ushiroda S, Maruyama Y (2001). Continuous observation of superoxide generation in an in-situ ischemia-reperfusion rat lung model. Jpn Circ J.

[B8] Weissmann N, Grimminger F, Olschewski A, Seeger W (2001). Hypoxic pulmonary vasoconstriction: a multifactorial response?. Am J Physiol Lung Cell Mol Physiol.

[B9] Zhang H, Slutsky AS, Vincent JL (2000). Oxygen free radicals in ARDS, septic shock and organ dysfunction. Intensive Care Med.

[B10] Fishman AP (1976). Hypoxia on the pulmonary circulation. How and where it acts. Circ Res.

[B11] Voelkel NF (1986). Mechanisms of hypoxic pulmonary vasoconstriction. Am Rev Respir Dis.

[B12] Sylvester JT (2001). Hypoxic pulmonary vasoconstriction: a radical view. Circ Res.

[B13] Archer SL, Huang J, Henry T, Peterson D, Weir EK (1993). A redox-based O2 sensor in rat pulmonary vasculature. Circ Res.

[B14] Archer SL, Nelson DP, Weir EK (1989). Simultaneous measurement of O2 radicals and pulmonary vascular reactivity in rat lung. J Appl Physiol.

[B15] Chandel NS, Maltepe E, Goldwasser E, Mathieu CE, Simon MC, Schumacker PT (1998). Mitochondrial reactive oxygen species trigger hypoxia-induced transcription. Proc Natl Acad Sci U S A.

[B16] Chandel NS, McClintock DS, Feliciano CE, Wood TM, Melendez JA, Rodriguez AM, Schumacker PT (2000). Reactive oxygen species generated at mitochondrial complex III stabilize hypoxia-inducible factor-1 alpha during hypoxia: a mechanism of O2 sensing. J Biol Chem.

[B17] Chandel NS, Schumacker PT (2000). Cellular oxygen sensing by mitochondria: old questions, new insight. J Appl Physiol.

[B18] Marshall C, Mamary AJ, Verhoeven AJ, Marhall BE (1996). Pulmonary artery NADPH-oxidase is activated in hypoxic pulmonary vasoconstriction. Am J Respir Cell Mol Biol.

[B19] Waypa GB, Chandel NS, Schumacker PT (2001). Model for hypoxic pulmonary vasoconstriction involving mitochondrial oxygen sensing. Circ Res.

[B20] Waypa GB, Schumacker PT (2002). O(2) sensing in hypoxic pulmonary vasoconstriction: the mitochondrial door re-opens. Respir Physiol Neurobiol.

[B21] Weissmann N, Tadic A, Hanze J, Rose F, Winterhalder S, Nollen M, Schermuly RT, Ghofrani HA, Seeger W, Grimminger F (2000). Hypoxic vasoconstriction in intact lungs: a role for NADPH oxidase- derived H(2)O(2)?. Am J Physiol Lung Cell Mol Physiol.

[B22] Munzel T, Afanas'ev IB, Kleschyov AL, Harrison DG (2002). Detection of superoxide in vascular tissue. Arterioscler Thromb Vasc Biol.

[B23] Zou L, Clanton TL (2002). Detection of reactive oxygen and nitrogen species in tissues using redox-sensitive fluorescent probes. Methods Enzymol.

[B24] Archer SL, Nelson DP, Weir EK (1989). Detection of activated O2 species in vitro and in rat lungs by chemiluminescence. J Appl Physiol.

[B25] Brubacher JL, Bols NC (2001). Chemically de-acetylated 2',7'-dichlorodihydrofluorescein diacetate as a probe of respiratory burst activity in mononuclear phagocytes. J Immunol Methods.

[B26] Sanders SP, Bassett DJ, Harrison SJ, Pearse D, Zweier JL, Becker PM (2000). Measurements of free radicals in isolated, ischemic lungs and lung mitochondria. Lung.

[B27] Katz SA, Venkatachalam M, Crouch RK, Heffner JE, Halushka PV, Wise WC, Cook JA (1988). Catalase pretreatment attenuates oleic acid-induced edema in isolated rabbit lung. J Appl Physiol.

[B28] Slepneva IA, Glupov VV, Sergeeva SV, Khramtsov VV (1999). EPR detection of reactive oxygen species in hemolymph of Galleria mellonella and Dendrolimus superans sibiricus (Lepidoptera) larvae. Biochem Biophys Res Commun.

[B29] Dikalov S, Grigor'ev IA, Voinov M, Bassenge E (1998). Detection of superoxide radicals and peroxynitrite by l-hydroxy-4-phosphonooxy-2,2,6,6-tetramethylpiperidine: quantification of extracellular superoxide radicals formation. Biochem Biophys Res Commun.

[B30] Dikalov S, Skatchkov M, Bassenge E (1997). Spin trapping of superoxide radicals and peroxynitrite by l-hydroxy-3-carboxy-pyrrolidine and l-hydroxy-2,2,6, 6-tetramethyl-4-oxo-piperidine and the stability of corresponding nitroxyl radicals towards biological reductants. Biochem Biophys Res Commun.

[B31] Weissmann N, Grimminger F, Walmrath D, Seeger W (1995). Hypoxic vasoconstriction in buffer-perfused rabbit lungs. Respir Physiol.

[B32] Weissmann N, Winterhalder S, Nollen M, Voswinckel R, Quanz K, Ghofrani HA, Schermuly RT, Seeger W, Grimminger F (2001). NO and reactive oxygen species are involved in biphasic hypoxic vasoconstriction of isolated rabbit lungs. Am J Physiol Lung Cell Mol Physiol.

[B33] Weissmann N, Akkayagil E, Quanz K, Schermuly RT, Ghofrani HA, Fink L, Hanze J, Rose F, Seeger W, Grimminger F (2004). Basic features of hypoxic pulmonary vasoconstriction in mice. Respir Physiol Neurobiol.

[B34] Dikalov S, Skatchkov M, Bassenge E (1997). Quantification of peroxynitrite, superoxide, and peroxyl radicals by a new spin trap hydroxylamine l-hydroxy-2,2,6,6-tetramethyl-4-oxo-piperidine. Biochem Biophys Res Commun.

[B35] Dikalov S, Skatchkov M, Fink B, Bassenge E (1997). Quantification of superoxide radicals and peroxynitrite in vascular cells using oxidation of sterically hindered hydroxylamines and electron spin resonance. Nitric Oxide.

[B36] Rosen GM, Finkelstein E, Rauckman EJ (1982). A method for the detection of superoxide in biological systems. Arch Biochem Biophys.

[B37] Navarro JA, Granadillo VA, Rodriguez-Iturbe B, Garcia R, Salgado O, Romero RA (1991). Removal of trace metals by continuous ambulatory peritoneal dialysis after desferrioxamine B chelation therapy. Clin Nephrol.

[B38] Misra HP (1979). Reaction of copper-zinc superoxide dismutase with diethyldithiocarbamate. J Biol Chem.

[B39] Samuni A, Goldstein S, Russo A, Mitchell JB, Krishna MC, Neta P (2002). Kinetics and mechanism of hydroxyl radical and OH-adduct radical reactions with nitroxides and with their hydroxylamines. J Am Chem Soc.

[B40] Urbanski NK, Beresewicz A (2000). Generation of *OH initiated by interaction of Fe2+ and CU+ with dioxygen; comparison with the Fenton chemistry. Acta Biochim Pol.

[B41] Winterbourn CC (1995). Toxicity of iron and hydrogen peroxide: the Fenton reaction. Toxicol Lett.

[B42] Ishikawa M (1993). Oxygen radicals-superoxide dismutase system and reproduction medicine. Nippon Sanka Fujinka Gakkai Zasshi.

[B43] Vig E, Gabrielak T, Leyko W, Nemcsok J, Matkovics B (1989). Purification and characterization of Cu, Zn-superoxide dismutase from common carp liver. Comp Biochem Physiol B.

[B44] Ross AH, McConnell HK (1975). Permeation of a spin-label phosphate into the human erythrocyte. Biochemistry.

[B45] Swartz HM, Sentjurc M, Morse PD (1986). Cellular metabolism of water-soluble nitroxides: effect on rate of reduction of cell/nitroxide ratio, oxygen concentrations and permeability of nitroxides. Biochim Biophys Acta.

[B46] Paky A, Michael JR, Burke-Wolin TM, Wolin MS, Gurtner GH (1993). Endogenous production of superoxide by rabbit lungs: effects of hypoxia or metabolic inhibitors. J Appl Physiol.

[B47] Savineau JP, Marthan R (1997). Modulation of the calcium sensitivity of the smooth muscle contractile apparatus: molecular mechanisms, pharmacological and pathophysiological implications. Fundam Clin Pharmacol.

[B48] Zhang AY, Yi F, Teggatz EG, Zou AP, Li PL (2004). Enhanced production and action of cyclic ADP-ribose during oxidative stress in small bovine coronary arterial smooth muscle. Microvasc Res.

[B49] Bailey SR, Mitra S, Flavahan S, Flavahan NA (2005). Reactive oxygen species from smooth muscle mitochondria initiate cold-induced constriction of cutaneous arteries. Am J Physiol Heart Circ Physiol.

[B50] Gryglewski RJ, Palmer RM, Moncada S (1986). Superoxide anion is involved in the breakdown of endothelium-derived vascular relaxing factor. Nature.

[B51] Archer SL, Reeve HL, Michelakis E, Puttagunta L, Waite R, Nelson DP, Dinauer MC, Weir EK (1999). O2 sensing is preserved in mice lacking the gp91 phox subunit of NADPH oxidase. Proc Natl Acad Sci U S A.

[B52] Voelkel NF, Allard JD, Anderson SM, Burke TJ (1999). cGMP and cAMP cause pulmonary vasoconstriction in the presence of hemolysate. J Appl Physiol.

[B53] Reeve HL, Michelakis E, Nelson DP, Weir EK, Archer SL (2001). Alterations in a redox oxygen sensing mechanism in chronic hypoxia. J Appl Physiol.

[B54] Brandes RP, Kreuzer J (2005). Vascular NADPH oxidases: molecular mechanisms of activation. Cardiovasc Res.

[B55] Schafer M, Schafer C, Ewald N, Piper HM, Noll T (2003). Role of redox signaling in the autonomous proliferative response of endothelial cells to hypoxia. Circ Res.

